# Fungal canker agents in apple production hubs of Iran

**DOI:** 10.1038/s41598-021-02245-8

**Published:** 2021-11-22

**Authors:** Abbas Nourian, Mina Salehi, Naser Safaie, Fatemeh Khelghatibana, Jafar Abdollahzadeh

**Affiliations:** 1 Department of Plant Pathology, Faculty of Agriculture, Tarbiat Modares University, Tehran, Iran; 2 Department of Plant Breeding and Genetics, Faculty of Agriculture, Tarbiat Modares University, Tehran, Iran; 3 Iranian Research Institute of Plant Protection, Agricultural Research, Education and Extension Organization (AREEO), Tehran, Iran; 4 Department of Plant Protection, Faculty of Agriculture, University of Kurdistan, Sanandaj, Iran

**Keywords:** Microbiology, Plant sciences

## Abstract

To identify apple canker casual agents and evaluate their pathogenicity and virulence in apple production hubs including West Azarbaijan, Isfahan and Tehran provinces; samples were collected from symptomatic apple trees. Pathogenic isolates on the detached branches were identified as *Cytospora cincta*, *Diplodia bulgarica*, *Neoscytalidium dimidiatum* and *Eutypa* cf. *lata*. *E.* cf. *lata* was reported as a potential apple canker causal agent in Iran for the first time based on the pathogenicity test on the detached branches, whereas it caused no canker symptoms in apple trees until 6 months after inoculation. Currently, *E.* cf. *lata* seems to be adapted to a single city. *C. cincta*, *D. bulgarica* and *N. dimidiatum* caused canker symptoms in apple trees. “*C. cincta*” and also “*C. cincta* and *N. dimidiatum*” were the most widespread and aggressive apple canker species, respectively, associated with apple canker in Iran. Therefore, they are considered to be the main threat to apple production in Iran and should be carefully monitored. Disease progress curve, area under the disease progress curve and optimum temperatures were determined for mentioned species. It is concluded that the establishment of each species occurs in appropriate areas and times in terms of the optimum temperature for their growth.

## Introduction

Apple (*Malus domestica* Borkh) is one of the most consumed and nutritious fruits in the world^1^. Iran is the fourth apple producer country after China, the United States and Turkey in terms of average production from 1994 to 2019^2^. It is noteworthy that Iran was the third apple producer country in the world until 2013^2^. While apple production has increased in some countries in recent years, Iran’s ranking dropped to fifth place in 2014, sixth in 2015 and seventh in 2016^2^. Among the different factors driving this downgrade, the fungal cankers are the most serious and important ones in apple orchards in Iran (personal communication with apple growers and agricultural extension experts). Recently, the phytosanitary status of the orchards has deteriorated because of the climate change and nutrient deficiency, and they increase the tree susceptibility to the fungal canker agents (personal communication with apple growers and agricultural extension experts). True opportunistic canker pathogens penetrate their hosts via wounds and cause dieback^3,4^. Indeed, these pathogens colonize and occlude the plant vascular system, thus disrupting water transport to infected trunks, shoots and branches, eventually leading in the death of the whole tree or even orchard failure^5^. It was stated that the greater importance should be attached to fungal trunk pathogens in Iran^6^.

Different fungal species have been isolated from apple tree displaying canker symptoms which mostly belong to Botryosphaeriaceae and Valsaceae including *Botryosphaeria dothidea* and *B. obtusa*^7^, *B. lutea*^8^, *Leucostoma cincta*^9^, *L. cinctum*^10–12^, *Cytospora rubescens*^13^, *C*. *leucostoma*, *C*. *personata*, *C. schulzeri* and *C. cincta*^14^, *C. ambiens*, *C. mali*^15^, *C. chrysosperma*^10–12^, *Valsa malicola*, *V. nivea*^16^, *Hendersonula toruloidea*^17^, *Diplodia intermedia*, *D. malorum*, *D. seriata* and *D. bulgarica*^18,19^, *Didymosphaeria rubi-ulmifolii*, *Schizophyllum commune*, *Didymella pomorum* and *Coniochaeta fasciculata*^4^, *Eutypa lata*^20–22^, *Neoscytalidium dimidiatum*^15^.

Fungal apple cankers have been the subject of much research worldwide, but few studies have focused on it in Iran, one of the largest apple producers in the world. Different fungal species could cause apple canker, although the main causal agent is unknown. The etiology of apple cankers in apple production hubs in Iran including West Azerbaijan (Urmia, Khoy and Salmas Counties), Isfahan (Semirom and Khomeyni Shahr Counties) and Tehran (Damavand County) was unclear. Therefore, the objective of this study was to investigate the etiology and also virulence of apple canker causal agents in planta.

## Results

### Isolation of apple canker causal agents

A total of 189 isolates were isolated from apple trees showing canker symptoms (Supplementary Table S1). The isolates were grouped based on the geographic area and morphotype (Supplementary Table S1), and then one representative was selected from each group (90 isolates) (Supplementary Tables S1 and S2).

### Pathogenicity test on the detached branches

**Figure 1:**
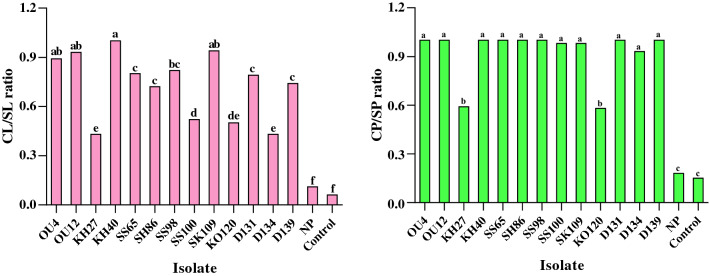
Canker length to branch length (CL/SL) and canker perimeter to branch perimeter (CP/SP) ratios in the preliminary pathogenicity/virulence tests on apple detached branches. Average values (quintuplicate) are given. *NP* non-pathogenic isolates displayed no symptom on apple detached branches. Means followed by the same letter are not significantly different according to LSD at 0.05 probability level.

The results showed that only 13 isolates of 90 tested isolates could cause the necrotic lesions on the detached branches (Fig. 1, Supplementary Tables S1–S3). The necrotic lesions appeared on the detached branches inoculated with pathogenic isolates 1 week after inoculation but no lesion was observed in the control or detached branches inoculated with non-pathogenic isolates even 4 weeks after inoculation (Fig. 1, Supplementary Table S3). The pathogenic isolates were re-isolated from lesions. The fungal isolates were significantly different in terms of canker length to stem length (CL/SL) and canker perimeter to stem perimeter (CP/SP) ratios (Fig. 1, Supplementary Table S3). Although, no significant differences were observed between different isolates of *D. bulgarica* (OU12 and KH40) and *C. cincta* (OU4, SS65, SH86, SS98, SS100, D131 D134 and D139) in terms of CP/SP (Fig. 1 and Supplementary Table S3). In this test, the highest and lowest virulence were recorded for “OU4, OU12, KH40, and SK109” and “KH27 and KO120” isolates, respectively (Fig. 1 and Supplementary Table S3).

### Identification and phylogenetic analysis

**Figure 2:**
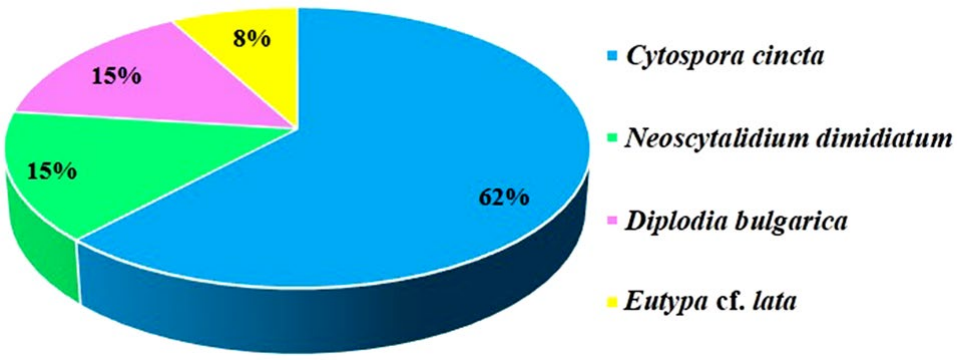
Frequency (%) of pathogenic isolates “*C. cincta* (OU4, SS65, SH86, SS98, SS100, D131, D134 and D139), *N. dimidiatum* (SK109 and KO120), *Diplodia bulgarica* (OU12 and KH40) and *Eutypa*. cf. *lata* (KH27)”.

The isolates causing necrotic lesions on the detached branches were re-isolated and identified by morphological characters and phylogenetic analysis of fungal internal transcribed spacer (ITS) fragments (ITS1-5.8S-ITS2) and translation elongation factor 1-α (TEF-1-α)/β-tubulin (BT) gene. Thirteen pathogenic isolates belonged to four species, *C. cincta* (62%), *N. dimidiatum* (15%), *Diplodia bulgarica* (15%) and *Eutypa*. cf. *lata* (8%) (Fig. 2).

**Figure 3:**
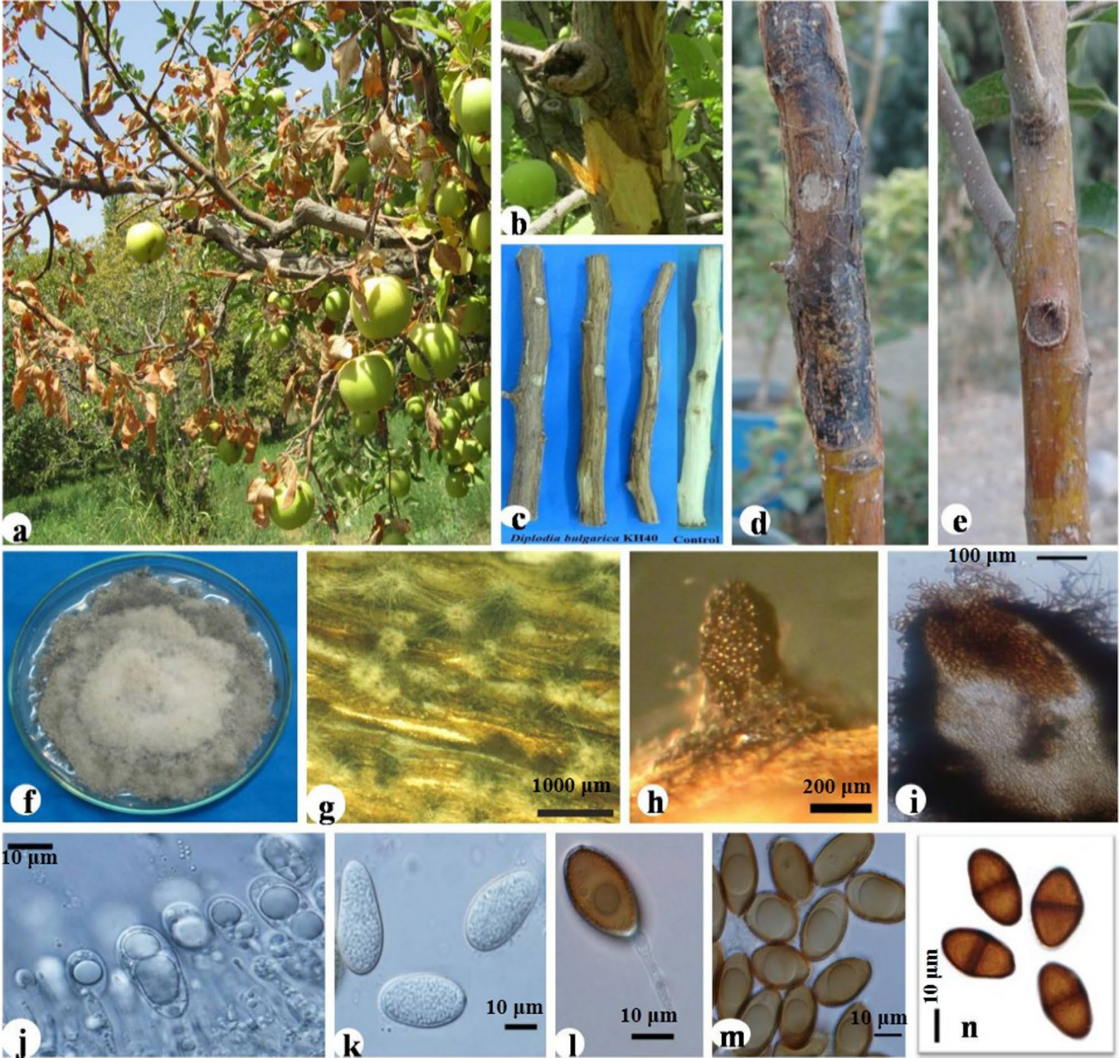
*Diplodia bulgarica* KH40. Symptoms of canker on infected apple trees in orchard (**a**,**b**), pathogenicity on detached branch and comparing it with control (**c**), pathogenicity on 2-year-old apple tree and comparing it with control (**d** = KH40 isolate and **e** = control), culture growing on potato dextrose agar (**f**), conidiomata developing on poplar twigs on water agar (**g**), conidia exuded from conidiomata developed on poplar twigs (**h**), vertical section through pycnidia (**i**), hyaline immature conidia developing on conidiogenous cells (**j**), hyaline aseptate conidia (**k**) brown mature conidium on conidiogenous cells (**l**), brown aseptate conidia (**m**), brown one-septateconidia (**n**).

**Figure 4:**
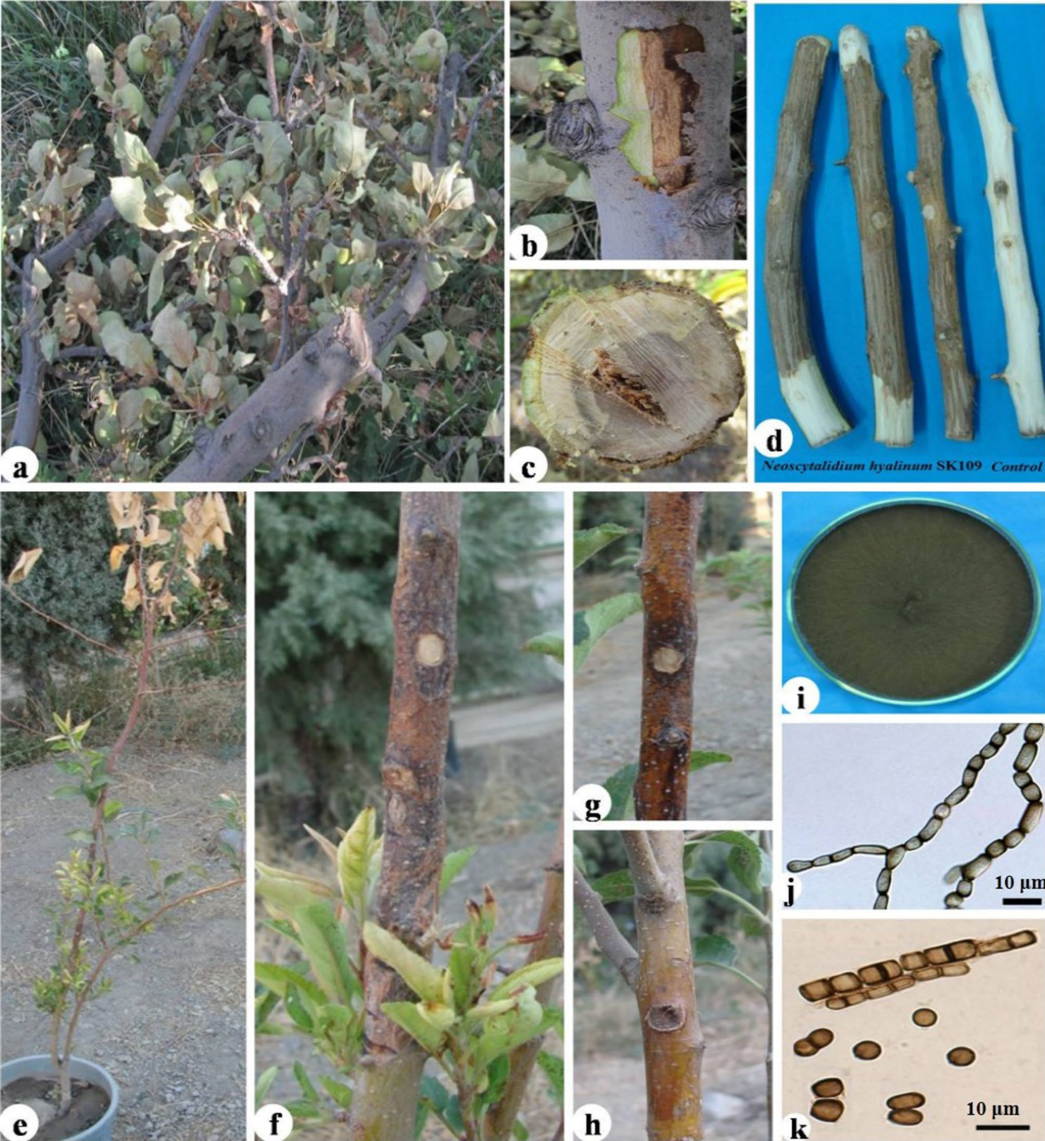
*Neoscytalidium dimidiatum* SK109. Symptoms of canker on infected apple trees in orchard (**a**–**c**), pathogenicity on detached branch and comparing it with control (**d**), pathogenicity on 2-year-old apple tree and comparing it with control (**e**–**g** = SK109 isolate and **h** = control), culture growing on potato dextrose agar (**i**), arthroconidia developing (**j**), mature 0–1-septate arthroconidia (**k**).

**Figure 5:**
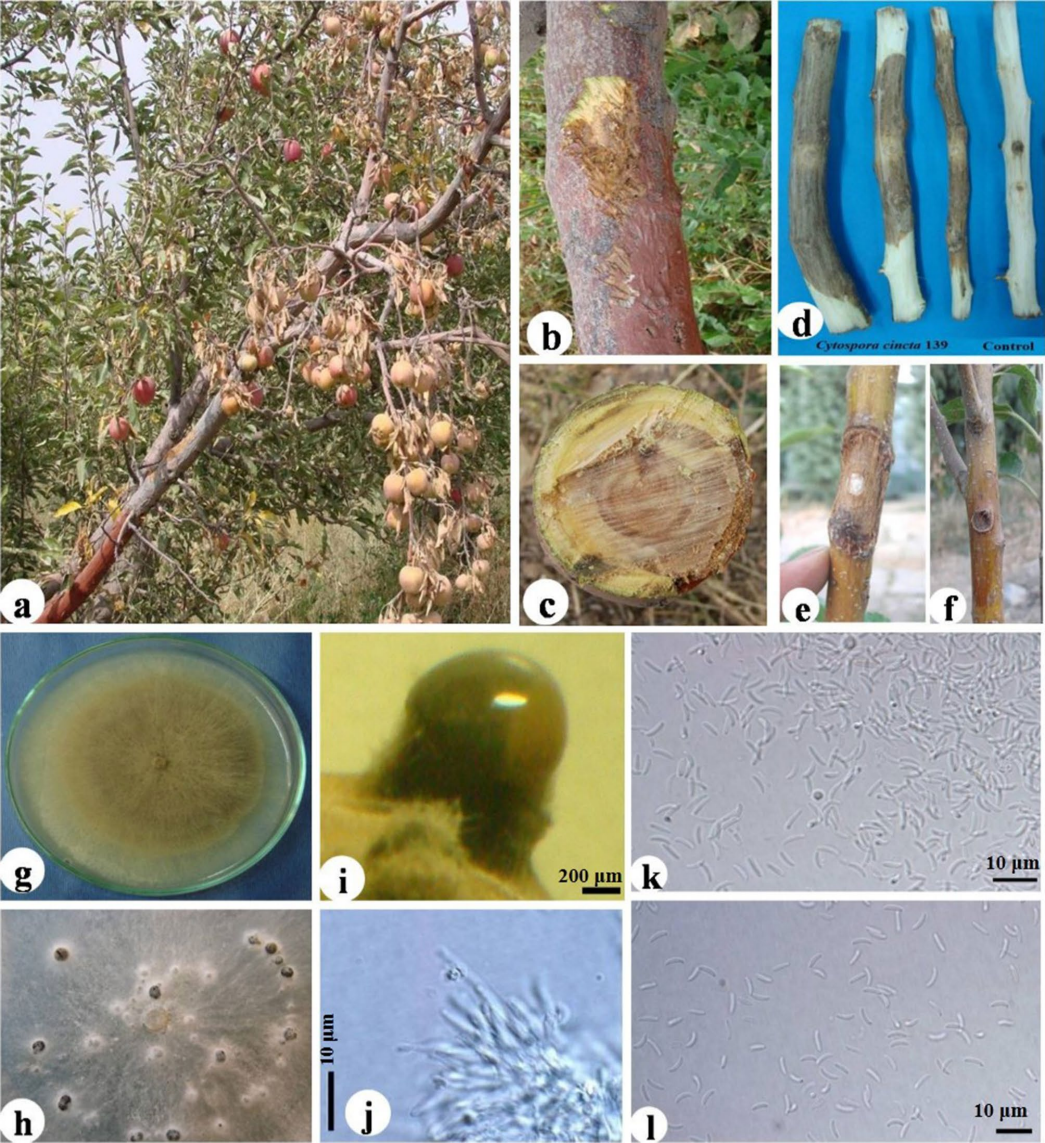
*Cytospora cincta* D139. Symptoms of canker on infected apple trees in orchard (**a**–**c**), pathogenicity on detached branch and comparing it with control (**d**), pathogenicity on the 2-year-old apple tree and comparing it with control (**e** = KH40 isolate, **f** = control), culture growing on potato dextrose agar (**g**), stromata developing on PDA (**h**), stromata developing on poplar twigs on water agar (**i**), conidiophores (**j**), hyaline allantoid conidia (**k**,**l**).

**Figure 6:**
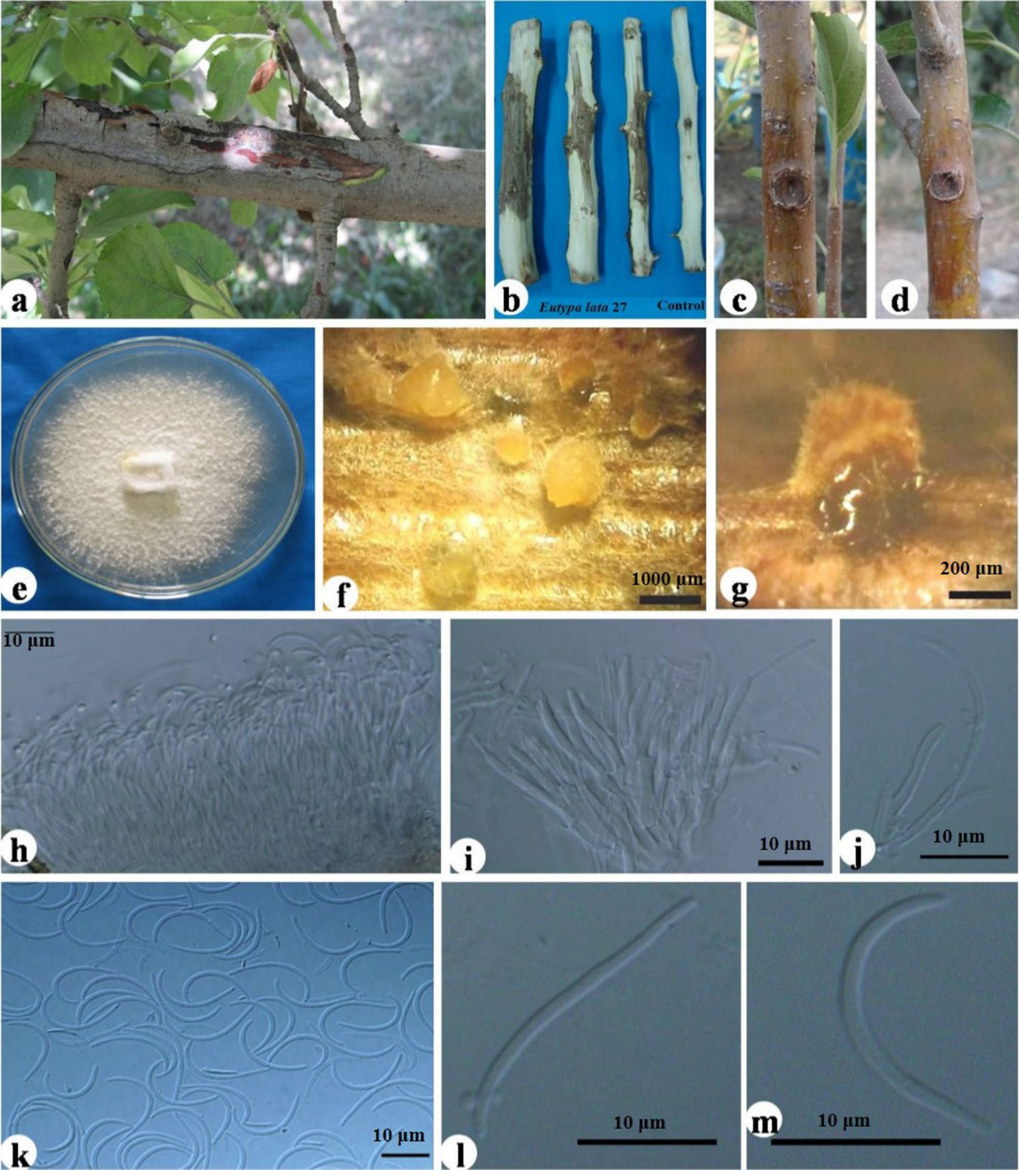
*Eutypa* cf. *lata* KH27. Symptoms of canker on infected apple trees in orchard (**a**), pathogenicity on detached branch and comparing it with control (**b**), pathogenicity on 2-year-old apple tree and comparing it with control (**c** = KH27 isolate, **d** = control), culture growing on potato dextrose broth (**e**), stromata developing on poplar twigs on water agar (**f**,**g**), conidia developing on conidiogenous cells conidiophores (**h**–**j**), hyaline filiform conidia (**k**–**m**).

The disease symptoms, pathogenicity and morphological features for each one of the species have been shown in Figs. 3, 4, 5 and 6.

***Cytospora cincta ***Sacc., (Teleomorph; *Leucostoma cinctum*).

The colonies of OU4, SS65, SH86, SS98, SS100, D131, D134 and D139 were initially white and turned yellowish at the surface and brown in the center with yellow margins in old cultures with age, also colony diameter was about 60 mm after 4 days on PDA at 25 °C. Stromata developed on Potato Dextrose Agar (PDA) after 6–8 weeks, which exuded cream to orange cirrhi. Conidiogenous cells hyaline and phialidic. Conidia hyaline, aseptate, allantoids with diameter (av. of 90 conidia); (3.9−) 4.8–5.6 (− 6.3) × (1−) 1.1–1.3 (− 1.5) μm (mean ± SD = 5.2 ± 0.5 × 1.2 ± 0.1, L/W ratio ± SD = 4.5 ± 0.6). These isolates (OU4, SS65, SH86, SS98, SS100, D131, D134 and D139) belonged to *Cytospora* genus based on the keys of Barnet and Hunter^23^. Identification of *Cytospora* species based on morphological features of fruiting bodies is impossible, therefore, the molecular method was used for their identification.

**Figure 7:**
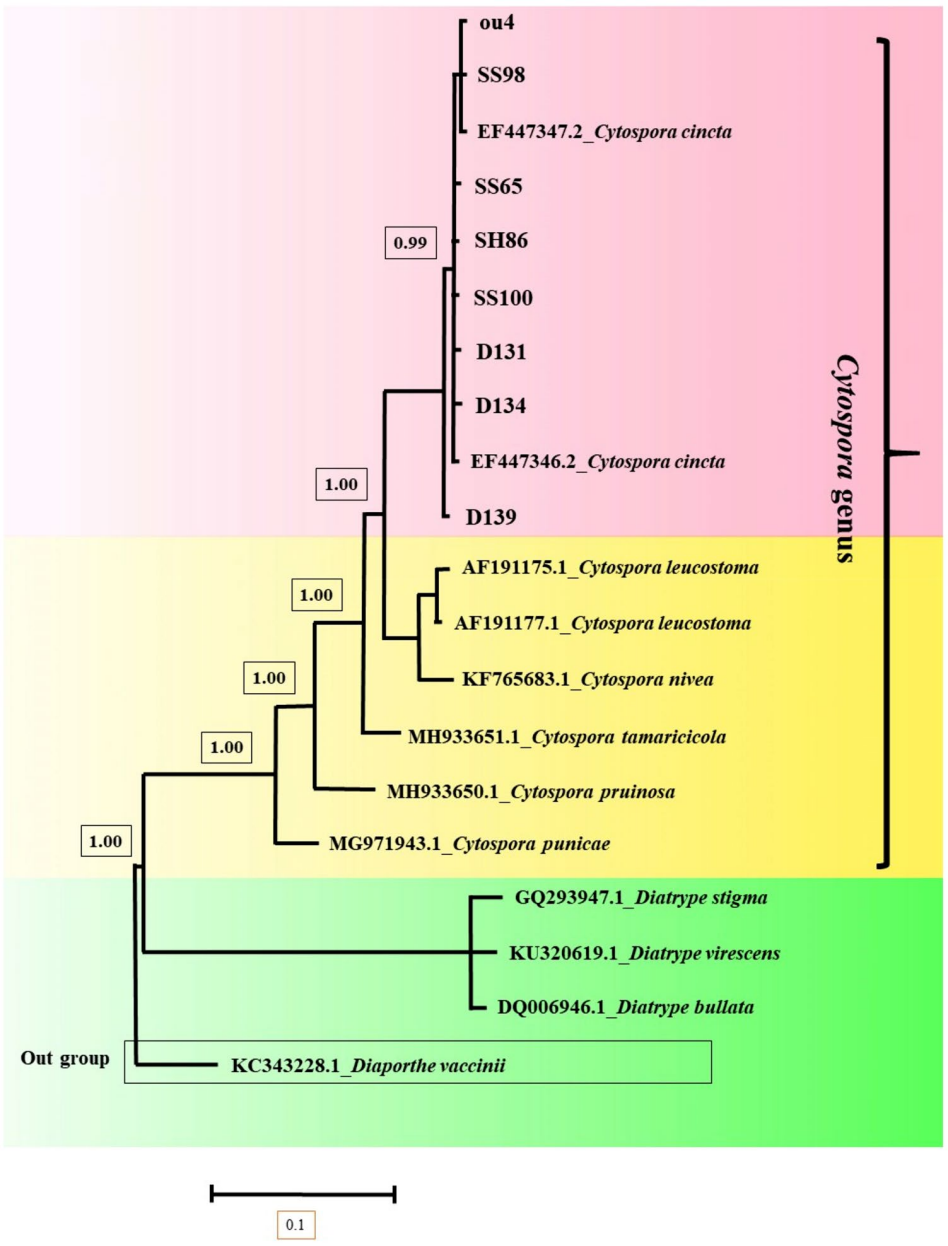
Bayesian 50% majority rule consensus tree deduced by means of ITS sequences of *Cytospora* spp. under the SYM + I + G model. The newly generated sequences (OU4, SS65, SH86, SS98, SS100, D131, D134 and D139) are in bold frameworks.

Phylogenetic analysis results showed that all pathogenic isolates of *Cytospora* were *Cytospora cincta* (*Leucostoma cinctum*) (Fig. 7).

***Diplodia bulgarica*** A.J.L. Phillips, J. Lopes & S.G. Bobev.

The colonies of OU12 and KH40 had aerial mycelium with rosette-shaped growth, colony color was initially white which turned to dirty green, in reverse side gray olive, colony diameter was about 70–80 mm after 5 days on PDA at 25 °C. Conidiomata were produced on poplar twigs on water agar (WA) under near-ultraviolet (NUV) after 2 weeks, the mature conidiomata were dark brown to black, globose to ovoid, conidiophore absent. Conidiogenous cells hyaline, holoblastic with conidiogenous ring, 9–18 × 2–6 μm. Conidia were initially hyaline, unicellular, ellipsoid to ovoid, became pale and dark brown after the discharge from conidiomata, one-septate with aging, smooth external and rough internal walls: (20−) 23–25.6 (− 29.5) × (12−) 12.9–14.1 (− 15.6) μm (mean ± SD = 24.3 ± 1.8 × 13.5 ± 0.8, L/W ratio ± SD = 1.8 ± 0.1) μm (av. of 48 conidia). These isolates (OU12 and KH40) were identified as *Diplodia bulgarica* according to species description by Phillips et al.^18^.

**Figure 8:**
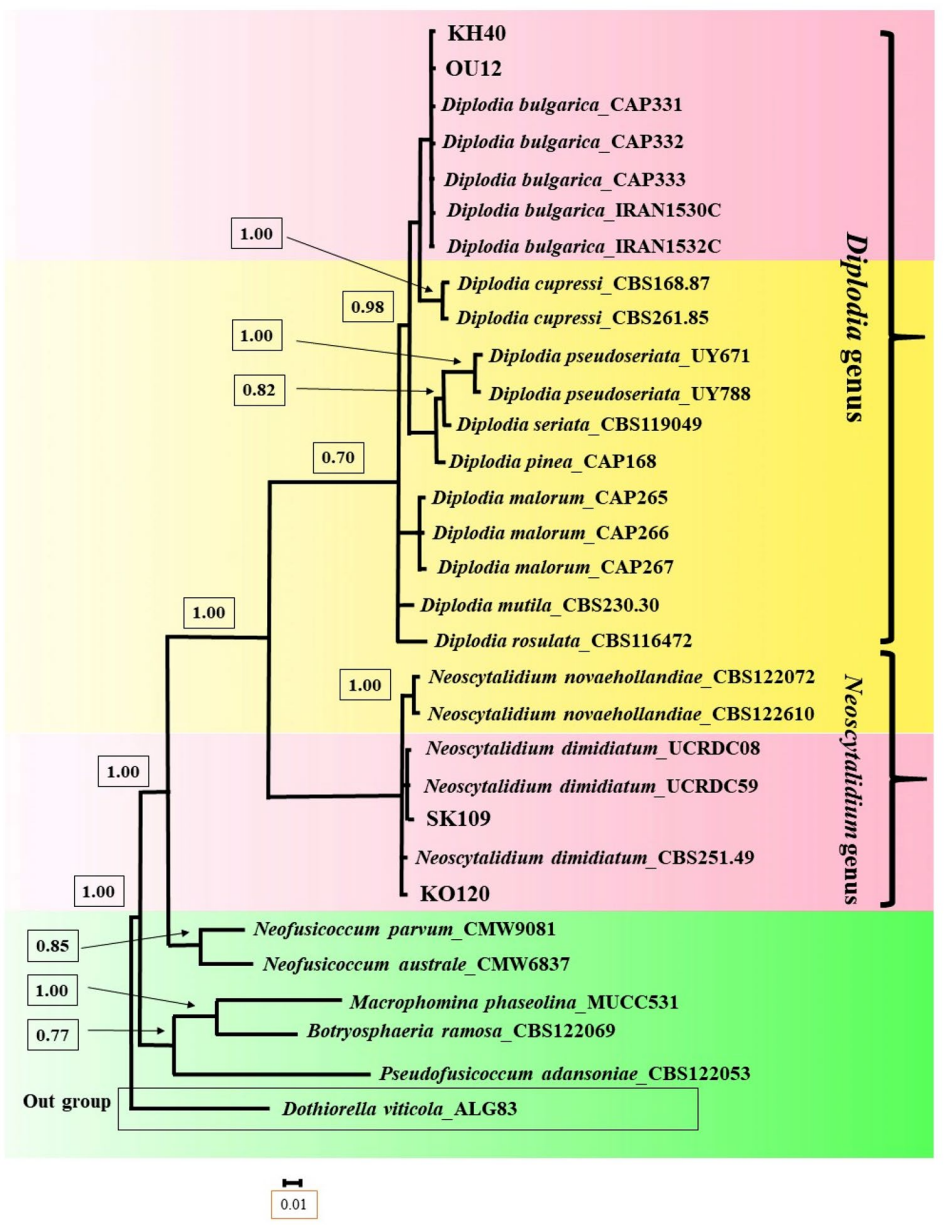
Bayesian 50% majority rule consensus tree deduced by means of ITS and TEF-1 α sequences of the *Diplodia and Neoscytalidium* spp. under the GTR + G model. The newly generated sequences (OU12, KH40, SK109 and KO120) are in bold frameworks.

Molecular analysis confirmed the results of morphological identification (Fig. 8).

***Neoscytalidium dimidiatum*** (C.K. Campbell & J.L. Mulder) A.J.L. Phillips, Groenewald & Crous.

Basionym: *Scytalidium dimidiatum* C.K. Campb. & J.L. Mulder.

 = *Torula dimidiata* Penz.

≡ *Scytalidium dimidiatum* (Penz.) B. Sutton & Dyko.

≡ *Fusicoccum dimidiatum* (Penz.) D.F. Farr.

The colonies of SK109 and KO120 displayed uniform radial growth, initially white and gradually olivaceous and finally dark, also the colony attained a diameter of about 90 mm after three days on PDA at 25 °C. The conidiomata were not produced on poplar twigs on water agar (WA) under NUV or PDA after 6-months. The aerial mycelium became arthrospore with age. The arthrospores were initially pale olive and eventually dark brown, thick-walled, 0–1-septate with age. One-septate arthrospores usually cylindrical with flat ends and rarely ellipse with the size (av. of 58 conidia): (6.1−) 7.7–9.6 (− 11.6) × (2.7−) 3.7–4.9 (− 6) μm (mean ± SD = 8.7 ± 1.2 × 4.3 ± 0.7, L/W ratio ± SD = 2.1 ± 0.4). The non-septate arthrospores usually spherical with diameter (av. of 46 conidia): (3.2−) 4.4–5.8 (− 7.2) μm (mean ± SD = 5.1 ± 0.9). According to the results of Nattras^17^, Sutton and Dyko^24^, Crous et al.^25^ and Phillips et al*.*^19^, these species (SK109 and KO120) were identified as *Neoscytalidium dimidiatum*.

The molecular analysis confirmed the result of the morphological identification (Fig. 8).

***Eutypa cf. lata*** (Pers.) Tul. & C. Tul.

The colonies of KH27 were white at the surface, aerial mycelium partly fluffy, in reverse side white, colony diameter was about 40 mm after 4 days on PDA at 25 °C. No stromata developed on PDA but was produced on poplar twigs on WA under NUV, stromata were dark after 6-weeks and exuded yellow and slimy cirrhi. Conidia hyaline, aseptate, filiform, curved and with the size (av. of 35 conidia); (19−) 21.2–24.1 (− 26) × (1.1−) 1.3–1.5 (− 1.6) μm (mean ± SD = 22.8 ± 1.8 × 1.4 ± 0.1, L/W ratio ± SD = 16.9 ± 2.3). Isolate “KH27” belonged to *Eutypa* genus according to the identification key for Diatrypaceae by Rappaz^26^. Since the identification of *Eutypa* spp. is based on sexual fruiting bodies on infected tissues and there were no fruiting bodies in sampling time, therefore, *Eutypa* sp. was completely identified based on molecular studies.

**Figure 9:**
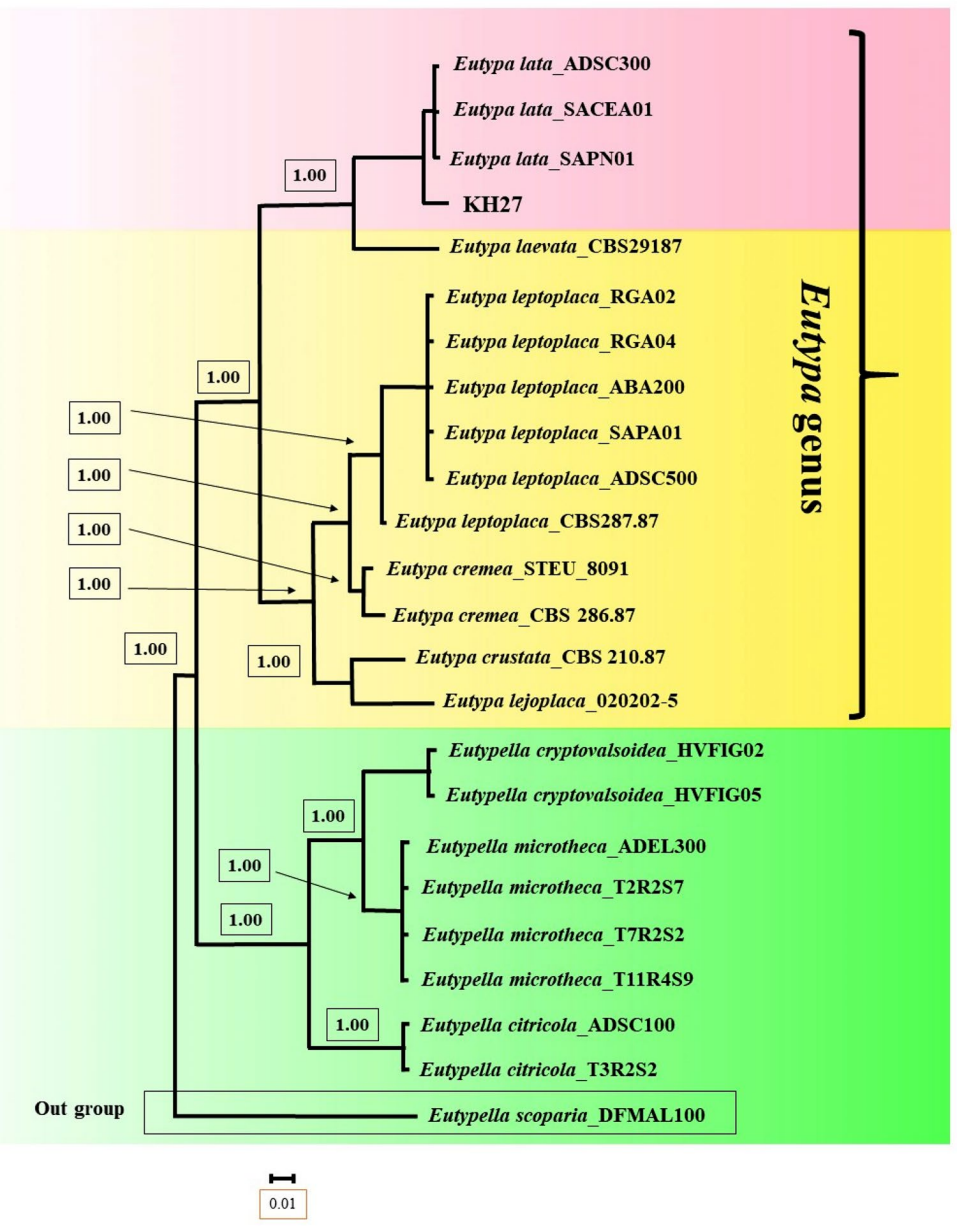
Bayesian 50% majority rule consensus tree deduced by means of ITS and β-tubulin sequences of the *Eutypa* sp. under the GTR + G model. The newly generated sequence (KH27) is in bold frameworks.

Phylogenetic analysis showed that *Eutypa* sp. KH27 was placed in clade *E. lata* and *E. laevata* (Fig. 9). Therefore, this species was identified as *E.* cf. *lata* based on molecular and morphological results. According to Rappaz^26^, *E. lata* and *E. laevata* resemble each other, and they are only different in conidia size and host range; *E. lata* (Conidia size; 20–30 × 1–1.5 and a wide host range of dicotyledonous plants) and *E. laevata* (Conidia size; 30–60 × 1–1.2 and host range: Salicaceae).

The partial sequences of ITS rDNA obtained from *C. cincta* OU4, SS65, SH86, SS98, SS100, D131, D134 and D139, *D. bulgarica* OU12 and KH40, *N. dimidiatum* SK109 and KO120 and also *E.* cf. *lata* KH27 were deposited in GenBank (NCBI) under the accession numbers “MZ266626, MZ266627, OK255702, MZ266628, OK255701, OK255700, OK255704, MZ266629, OK255703, MZ266618, MZ266620, OK255705, and MZ266630”. Also, the partial sequences of TEF-1-α gene obtained from *D. bulgarica* OU12 and KH40, *N. dimidiatum* SK109 and KO120, and also partial β-tubulin gene sequence of *E.* cf. *lata* KH27 were deposited in NCBI under the accession numbers “OK287404, OK287405, OK287406, OK287407 and OK287408”.

### Symptoms of different species in orchards

The symptoms caused by different species were determined in the orchards. It is noteworthy that the canker symptoms caused by *C. cincta*, *Diplodia bulgarica*, *N. dimidiatum* and *E.* cf. *lata* were different in appearance.

Symptoms of *C. cincta* were twig and branch dieback and necrotic bark. *Cytospora* canker is characterized by diffuse resinous branch cankers, with fruiting bodies of the causal fungi usually forming on infected parts.

*D. bulgarica* caused stem and trunk cankers, bark discoloration and scaling-off of the bark.

Symptoms of cankers caused by *N. dimidiatum* included bark lesions, discoloration of xylem tissues, longitudinal wood necrosis and extensive spore production as black powder under bark, spur and shoot blight.

Apple trees infected by *E.* cf. *lata* displayed branch and scaffold dieback, with dead leaves still attached indicating rapid death during the season.

Only *N. dimidiatum* was identified in Khomeini Shahr County (Isfahan Province). But both species “*N. dimidiatum* and *C. cincta*” were identified in Semirom County of Isfahan Province (Supplementary Table S1, Figs. 7 and 8). The severity of canker caused by *C. cincta* was similar to that with *N. dimidiatum* (Data not shown).

In Damavand, only *C. cincta* was identified (Supplementary Table S1 and Fig. 7).

In Urmia County (West Azerbaijan Province), *C. cincta* and *D. bulgarica* were identified (Supplementary Table S1 and Figs. 7 and 8). The severity of canker caused by *C. cincta* was higher than that with *D. bulgarica* (Data not shown).

In Khoy County (West Azerbaijan Province), *D. bulgarica* and *E.* cf. *lata* species were identified (Supplementary Table S1 and Figs. 8 and 9). The severity of canker caused by *D. bulgarica* was higher than with *E.* cf. *lata* (Data not shown).

### Pathogenicity test on 2-year-old apple trees

**Figure 10:**
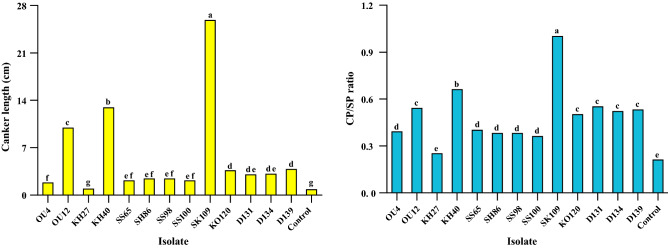
Canker length (CL) and canker perimeter to stem perimeter (CP/SP) ratio in the pathogenicity/virulence tests of *Cytospora cincta* (OU4, SS65, SH86, SS98, SS100, D131, D134 and D139), *Diplodia bulgarica* (OU12 and KH40), *Neoscytalidium dimidiatum* (SK109 and KO120) and *Eutypa* cf. *lata* (KH27) on 2-year-old apple trees. Average values (triplicate) are given. Means followed by the same letter are not significantly different according to LSD at 0.05 probability level.

The symptoms were evaluated 6 months after inoculation (26 May to 24 November 2013). The result showed that *N. dimidiatum*, *D. bulgarica* and *C. cincta* caused canker symptoms, but *E*. cf. *lata* did not cause canker symptoms on 2-years-old apple trees (Fig. 10). Comparison of Means (LSD, 0.05 level) for both parameters “CL and CP/SP” indicated a significant difference between pathogenic isolates except *Eutypa* cf. *lata* (KH27) as compared to the control (Fig. 10 and Supplementary Table S3). Also, the various species differently affected CL and CP/SP parameters. In this test, the highest and the lowest virulence were recorded for “*N. dimidiatum* SK109” and “*C. cincta* OU4, SS65, SH86, SS98 and SS100”, respectively (Fig. 10 and Supplementary Table S3).

### Disease progress curve

**Figure 11:**
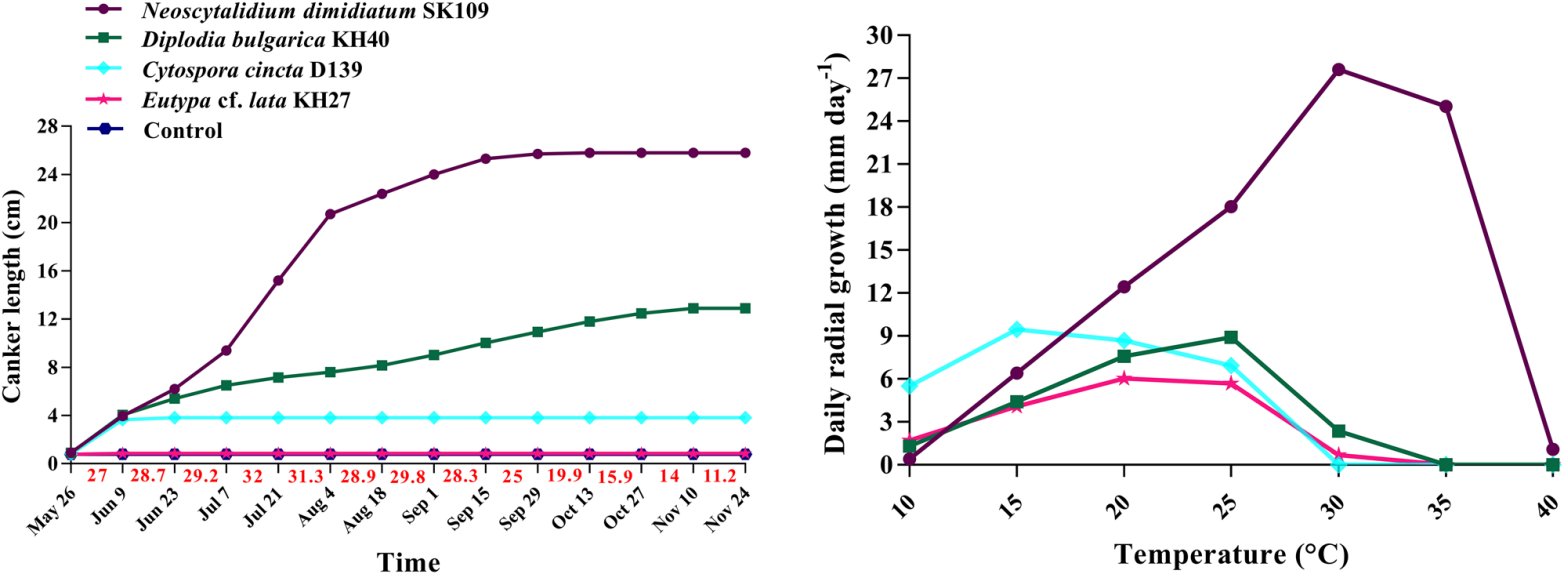
Canker progress curves of different species in the pathogenicity test on 2-year-old apple trees, Temperature is in red, and effect of temperature on daily radial growth of different species on potato dextrose agar. Average values (triplicate) are given.

Disease progress curves (DPCs) were plotted for the pathogenic isolates (Fig. 11 and Supplementary Fig. S1, and Supplementary Table S4). DPC of *N. dimidiatum* SK109 can be divided into three sections. In the first and second sections, the slope of the curve is increasing while the increment of the curve slope in the second section is higher than the first one. Indeed, ascending temperature resulted in ascending disease progresses, and the curve slope increased, so that, it is the highest level from July 7 to Aug 4. The temperature of this section was about 31.6 °C which the upper portion of the canker was dried. The curve slope in the third section is lower than the first and second ones. Descending temperatures led to descending the curve slope until October 13, which was stopped (≤ 19.9 °C) (Fig. 11).

DPC of *D. bulgarica* KH40 can be divided into two sections. In the first section, the curve slope is higher than the second one (May 26 to Jun 9 with temperature about 27 °C). The curve slope (CL development) in the second section is lower as compared to the first ones, and disease progress was stopped on Nov 10 (≤ 14 °C) (Fig. 11).

DPC of *C. cincta* D139 consists of one section, ascending temperature led to ascending apple canker progress until June 9 (27 °C) (Fig. 11). The progress of apple canker caused by *C. cincta* D139 was stopped on June 9 (≥ 27 °C) (Fig. 11).

It is noteworthy that canker symptoms in apple trees inoculated with *E*. cf. *lata* KH27 was not observed. Therefore, the progress curve of apple canker caused by *E*. cf. *lata* KH27 was linear, similar to control (Fig. 11).

**Figure 12:**
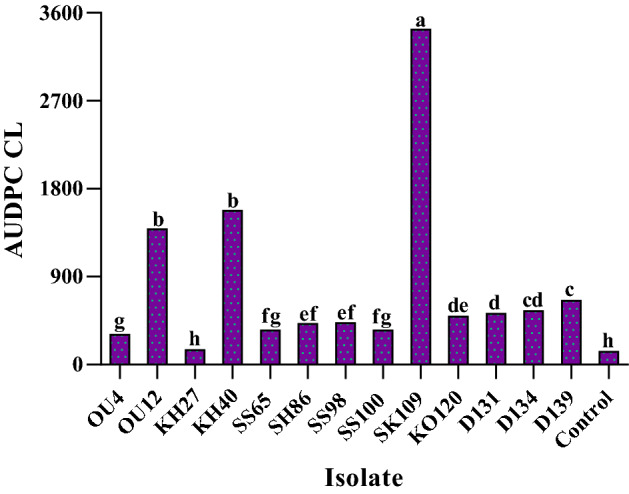
Area under disease progress curve (AUDPC**)** for canker length (CL) in the pathogenicity/virulence tests on 2-year-old apple trees. Average values (triplicate) are given. Means followed by the same letter are not significantly different according to LSD at 0.05 probability level.

Area under disease progress curve (AUDPC) was calculated for CL parameter. AUDPC of pathogenic isolates [*C. cincta* (OU4, SS65, SH86, SS98, SS100, D131, D134 and D139), *D. bulgarica* (OU12, KH40) and *N. dimidiatum* (SK109 and KO120)] except *Eutypa* cf. *lata* (KH27) showed the significant differences as compared to the control (Fig. 12 and Supplementary Table S3). *E.* cf. *lata* displayed no significant with the control in terms of AUDPC for CL (Fig. 12 and Supplementary Table S3). The highest and the lowest AUDPC were recorded for *N. dimidiatum* (SK109) and *C. cincta* (OU4, SS65 and SS100), respectively (Fig. 12 and Supplementary Table S3).

### Effect of temperature on fungal growth

Daily radial growths of 13 isolates causing apple cankers were presented in Fig. 11 and Supplementary Fig. S2. The optimum growth temperature of *C. cincta* D139, *D. bulgarica* KH40, *N. dimidiatum* SK109 and *E.* cf. *lata* KH27 were 15–20, 25, 30 and 20–25 °C, respectively (Fig. 11). As shown by Fig. 11 and Supplementary Fig. S2, *C. cincta*, *D. bulgarica* and *E.* cf. *lata* were not able to grow above 35 °C, whereas *N. dimidiatum* could grow up to 40 °C. At 10–15 °C, *C. cincta* D139 grew faster than the three other species (*D. bulgarica* KH40, *N. dimidiatum* SK109 and *E.* cf. *lata* KH27) and was able to grow even below 10 °C (Fig. 11). Accordingly, *D. bulgarica* and *E.* cf. *lata* are mesophile, *N. dimidiatum* and *C. cincta* are thermophile and psychrophile, respectively.

## Discussion

Four species, isolated from the apple trees displaying canker symptoms in Iran, were identified based on morphological characters and phylogenetic analysis. They belonged to four different genera in three different families, *D. bulgarica* and *N. dimidiatum* (Botryosphaeriaceae), *C. cincta* (Valcaceae) and *E.* cf. *lata* (Diatrypaceae). All identified species caused canker symptoms on the detached branches in the pathogenicity tests. *D. bulgarica*, *N. dimidiatum* and *C. cincta* caused canker symptoms on 2-year-old apple trees in the pathogenicity test. All the isolates of *C. cincta* (OU4, SS65, SH86, SS98, SS100, D131, D134 and D139) displayed a good growth rate at 15–25 °C (Fig. 11 and Supplementary Fig. S2). It should be noted that the temperature in the detached branch test was 25 °C, but the field temperature was more than 25 °C from May 26 to September 15 (Fig. 11). This could explain why all the isolates of *C. cincta* displayed more virulence on apple detached branches than that on 2-year-old trees. Also, *E*. cf. *lata* KH27 caused no canker symptoms until 6 months after inoculation. Accordingly, the optimum temperature for *E.* cf. *lata* KH27 was recorded 20–25 °C (Fig. 11), which was provided in the detached branch test, but the temperature in the pathogenicity on 2-year-old tree in the field was not optimal during 6 months of the experiment period except September 15–29 (Fig. 11). It can be the reason why *E.* cf. *lata* KH27 was not pathogenic on 2-year-old apple trees in the field.

*Eutypa lata* was reported as an apple canker causal agent^20,21,27^, and also its pathogenicity test was performed on the detached branches^22^, but this is the first attempt of the pathogenicity test of *E.* cf. *lata* on the apple tree. Pathogenicity test for *E. lata* was conducted on 2-year-old *Ribes rubrum* and the symptoms were observed 21 months after inoculation^28^. Also, the pathogenicity test of this species was performed on rooted cuttings of grapevine under greenhouse conditions (with a temperature about 22 °C) and the pathogenicity was confirmed 54 months after inoculation^29^. According to the results of the pathogenicity test of *E. lata* on grapevine^28^ and *Ribes rubrum*^29^, the pathogenicity test for this species should be done in controlled conditions (greenhouse and growth chamber) because disease symptoms appeared at a very long time after inoculation at the optimum temperature.

In this study, the disease symptoms did not appear in the apple trees inoculated with *E*. cf. *lata* until 6 months after inoculation under field conditions, whereas its pathogenicity and virulence were confirmed on the detached branch. *E.* cf. *lata* was reported as an apple potential canker causal agent in Iran based on pathogenicity test on the detached branches for the first time. Nevertheless, the pathogenicity test on the detached branch presents useful evidence, the field tests on apple trees should be conducted to confirm the pathogenicity and also determine disease severity in orchards.

*Cytospora cincta* (anamorph of *L. cinctum*) has been previously reported as canker causal agent in different regions of Iran^10–12^. It is noticed that only the pathogenicity of isolates from Karaj-Iran had been demonstrated^10^. Besides, the pathogenicity of other species of *Cytospora* isolated from the apple trees displaying canker symptoms has not been confirmed. In the world, only the pathogenicity of *L. cinctum* in Michigan has been demonstrated^9^. In this study, *C. cincta* was reported for the first time from West Azerbaijan and Damavand-Tehran provinces of Iran.

*Diplodia bulgarica* had been reported on apple trees displaying canker symptoms in West Azarbaijan and Kermanshah provinces of Iran, and also its pathogenicity had been confirmed^30,31^. Also*, D. seriata* and *D. malorum* were reported as apple canker causal agents in West Azarbaijan and Kermanshah provinces of Iran^32,33^. In this study, *D. bulgarica* was the highest incidence in West Azarbaijan province (Data not shown).

Also, *N. dimidiatum* was reported as apple dieback agent in tropical and subtropical regions of Iran, Khuzestan, Kerman and Fars Provinces^15^. However, its pathogenicity has not been reported on apple trees. Also, this species has not been reported from the main apple-growing areas in Iran (cold regions). In this study, the pathogenicity of *N. dimidiatum* was confirmed on the apple detached branches and trees. Also, *N. dimidiatum* was reported from Isfahan province of Iran for the first time.

Despite the other studies, AUDPC was measured and compared for these species. Canker’s progress of *N. dimidiatum* (SK109) was more than other species (Fig. 11 and Supplementary Fig. S1, and Supplementary Table S3) because the growth rate of *N. dimidiatum* SK109 was higher than other species. The other reason is the growth optimum temperature for this species (about 30 °C, Fig. 11) provided during the pathogenesis. The progress of the canker caused by *C. cincta* D139 was stopped 2 weeks after inoculation (Jun 9) because the average temperature of the assessment region was 27.8 °C after this period which D139 grew slightly at temperatures above 25 °C (Fig. 11).

Two pathogenic species (*N. dimidiatum* and *C. cincta*) were identified in Isfahan province (Semirom and Khomeini Shahr counties) (Supplementary Table S1, Figs. 7 and 8). Only *N. dimidiatum* was identified in Khomeini Shahr County (Kooshk city). But both species “*N. dimidiatum* and *C. cincta*” were identified in Semirom County (Supplementary Table S1, Figs. 7 and 8). In Semirom county, *C. cincta* was more prevalent than *N. dimidiatum* (Data not shown). Indeed, *N. dimidiatum* species was identified only in one city (Komeh) which is southern part of Semirom County and has warmer climate. It is interesting that *C. cincta* was not found in Komeh city (Supplementary Table S1 and Fig. 7). The progress peak of apple canker caused by *N. dimidiatum* was recorded on July and August (Data not shown). Additionally, May, June and September were the progress peak of apple canker caused by *C. cincta* (Data not shown).

In Damavand, only *C. cincta* was identified (Supplementary Table S1 and Fig. 7). There was a difference between the isolates of *C. cincta* in terms of virulence and the most virulent isolates on 2-year-old apple trees were the isolates (D13, D134 and D139) of Damavand (Fig. 10 and Supplementary Table S1). May and September were apple canker progress peak in Damavand (Data not shown).

In Urmia County (West Azerbaijan Province), *C. cincta* and *D. bulgarica* were identified (Supplementary Table S1 and Figs. 7 and 8). The progress peak of canker caused by *C. cincta* occur on May, June and September (Data not shown). Also, the progress peak of canker caused by *D. bulgarica* was observed on July and August (Data not shown).

In Khoy County (West Azerbaijan Province), *D. bulgarica* and *E. lata* species were identified (Supplementary Table S1 and Figs. 7 and 8), which *E.* cf. *lata* is not considered as an important pathogen due to low disease progress. The progress peak of apple canker caused by *D. bulgarica* was observed from June to August (Data not shown).

It is concluded that the establishment of each species occurs in areas compatible with their growth, and also the highet progress in canker symptoms for each species occurs in appropriate times in regard to the optimum temperature for their growth (Supplementary Table S5).

Based on this study and the results of Proffer and Jones^9^, it is suggested that only *C. cincta* (*L. cinctum*) is an important pathogen among *Cytospora* spp. isolated from apple tree displaying canker symptoms. In this study, isolates of *C. cincta* were isolated from thick branches with high infection. Proffer and Jones^9^ isolated different fungal species from apple trees displaying canker symptoms in Michigan. They conducted the pathogenicity test for *V. malicola* (teleomorph *C. schulzeri*), *L. cinctum*, *B. stevensii* and *B. obtusa* and evaluated their symptoms 6 weeks after inoculation. The results showed that isolates of *L. cinctum*, *B. stevensii* and *B. obtusa* were able to make canker symptoms, while isolates of *V. malicola* caused no canker symptoms.

The current research presents the first in-depth study regarding the isolation, pathogenicity, virulence and phylogenetic analysis of fungal pathogens associated with apple canker in Iran. Given the spread of canker disease on apple as well as its economic importance in Iran, the identification of apple canker agents would pave the way for its integrated management including biological control with focusing on aggressive species and/or isolates. Besides, since different causal agents including *D. bulgarica*, *N. dimidiatum*, *C. cincta*, and *E.* cf. *lata* contribute to apple tree cankers, it is essential to take measures to manage their spread to new orchards. According to our results, *C. cincta* is the most widespread canker pathogens of apple in Iran. Additionally, *C. cincta* and *N. dimidiatum* is the most aggressive apple canker pathogens in Iran. Also, *N. dimidiatum* SK109 displayed the most virulence on Golden delicious cultivar, one of the most widely apple cultivar. Therefore, *C. cincta* and *N. dimidiatum* are considered to be a main threat to apple production in Iran and should be carefully monitored. Currently, *E.* cf. *lata* seems to be adapted to a single city (West Azerbaijan province-Khoy county-Firuraq city); but additional sampling will likely reveal its further geographical and host range. It is noteworthy that 77 of the tested isolates were not able to cause canker symptoms. A number of these isolates may be associated with apple canker and contribute to disease. Since apple trees are perennial plants, they may be infected with different fungal species inciting a complex disease in orchestrate with two or several fungal species. Meanwhile, additional pathogenic fungi may cause apple canker in Iran, remain to be investigated. Therefore, the further studies are still needed to identify apple canker causal agents and also discover their roles in disease establishment and severity in Iran.

## Materials and methods

### Sampling and fungal isolation

The survey was conducted in three major apple production provinces namely West Azerbaijan (Urmia, Khoy and Salmas Counties), Isfahan (Semirom and Khomeyni Shahr Counties) and Tehran (Damavand County). Seventeen sites (Ghafar Behi, Tala Tappeh, Shur Kand, Qaraguz-e Hajji Baba, Zaviyeh-e Hasan Khan, Vardan, Kharab, Tale Robah, Ayneh Varzan and Sarbandan villages, and also Firuraq, Tazeh Shahr, Hana, Komeh, Kooshk, Khomeyni Shahr and Absard cities) were selected from three provinces (Supplementary Table S1), and 10 trees were randomly selected from four orchards in each site From May to September 2012. It is noteworthy that sampling was performed from Golden Delicious and Red Delicious, the main planted cultivars in Iran. Symptomatic branches with 2 to 5 cm in diameter were cut and transported to the lab in paper bags. The samples were firstly disinfected by 70% ethanol, then bark was removed, and small pieces (5 × 5 mm) of wood tissue were dissected from the margin of the canker. The pieces were placed on Petri dishes containing PDA medium amended with chloramphenicol (200 mg/L). Emerging colonies were purified using hyphal tip culture on 2% water agar (WA).

### Plant materials

It is declared that 2-year-old apple trees (cv. Golden Delicious) were legally bought from local commercial fruit trees provider company named ITA-Sadra (http://itasadra.ir/?page_id=2196&lang=en) and all methods involving plant studies were performed in accordance with the relevant guidelines and regulations.

### Pathogenicity tests on detached branches

Firstly, 90 isolates were selected according to the geographic region and morphotype (Supplementary Tables S1 and S2). Then pathogenic isolates were detected based on the pathogenicity test on the detached branches^34^. For this purpose, the detached branches (20 cm long and 1.5 to 2 cm diameter) from 2-year-old apple trees (cv. Golden Delicious) were sealed by Parafilm at two both ends to avoid dehydration and surface-sterilized using 70% ethanol and inoculated in the middle after removing the cortex with a cork borer (5-mm diameter). Instantly a mycelium plug (5-mm diameter from 4-day-old cultures) of each isolate was placed on the wound in direct contact with vascular tissue, then wrapped using Parafilm. The control was inoculated with PDA plug. To keep the relative humidity high, the control and inoculated detached branches were placed on a plastic mesh plate in a plastic container covered with a moistened paper towel at the bottom and maintained at 25 °C. CL/SL and also CP/SP ratio were measured 4 weeks after inoculation. The experiment was conducted in a randomized complete block design (RCBD) with five replications.

### Identification of pathogenic isolates

#### Morphological identification

To induce sporulation, the isolates were cultured on WA medium having pieces of double-autoclaved and halved poplar twigs, then incubated under NUV light with a 12-h photoperiod at 23–27 °C for a suitable period ranging from 2 to 10 weeks^19^. The cultures were microscopically inspected at regular intervals during the incubation period. After emerging the conidiomata on poplar twigs, they were vertically cut using a sharp scalpel, and the sporogenous tissues were mounted on a microscope slide containing a drop of 100% lactic acid, then the characteristics of spores and other organs were recorded.

#### Molecular identification

For DNA extraction, the fungal isolates were cultured in 200 mL flasks containing 50 mL Potato Dextrose Broth (PDB) and kept on a rotary shaker at 120 rpm for 4–7 days at 25 °C. The mycelia were separated from the fluid by vacuum filtration on No. 1 Whatman filter paper, lyophilized, and stored at − 80 °C. DNA extraction was performed according to previous studies^35,36^. Primer pairs “ITS1/ITS4^37^, EF1-728F/EF1-986R^38^ and Bt2a/Bt2b^39^” were used to amplify nuclear ribosomal DNA internal transcribed spacer (ITS) region, translation elongation factor 1-α (TEF-1-α) and β-tubulin (BT), respectively. Polymerase chain reaction (PCR) mixtures (25 µl) consisted of 1 µl genomic DNA (~ 30 ng), 1 µl forward and reverse primers (10 pM), and 12.5 µl Premix Taq (TaKaRa Biotechnology Ltd., Japan), and 10.5 µl PCR quality water. PCR reaction programs were an initial denaturation at 94 °C for 3 min, followed by 30 cycles of denaturation (94 °C for 30 s), annealing (56 °C for 30 s), extension (72 °C for 1 min) and a final extension at 72 °C for 5 min. PCR products were analyzed by agarose gel electrophoresis and purified using a DNA gel extraction kit (Axygen Biotechnology Ltd., China). Purified PCR product was directly sequenced using the same primers by Bioneer (Shanghai, China).

Newly obtained sequences of *Eutypa* sp. (KH27), *Diplodia* sp. (KH40 and OU12), *Neoscytalidium* sp. (SK109 and KO120), and *Cytospora* sp. (OU4, SS65, SH86, SS98, SS100, D131, D134 and D139) together with the other sequences of related genus were already used by other studies^11,18,19,40–45^ were selected for phylogenetic analyses. The dataset was updated by investigations in the database for acquiring accurate sequences. The outgroup taxa for present dataset were taken based on previous studies^41,42,45^. All sequences were aligned using Q-INS-i algorithm of MAFFT version 7 (http://mafft.cbrc.jp/alignment/server/)^46^ and the online version of Gblocks 0.91b^47^ was applied to remove ambiguous parts of the alignment, with all three options (including allow smaller final blocks, allow gap positions within the final blocks, and allow less strict flanking position) for a fewer stringent selection (http://molevol.cmima.csic.es/castresana/Gblocks_server.html). The most suitable substitution model for dataset was chosen using Akaike information criterion (AIC) by means of PAUP ∗ /MrModeltest v2.2. A symmetrical model including a gamma distribution were picked out for rates with SYM + I + G for *Cytospora* with ITS analysis, and GTR + G for *Eutypa* with ITS and BT analysis, *Diplodia* and *Neoscytalidium* with ITS and TEF-1-α analysis. Bayesian inference (BI) was carried out by means of MrBayes v3.1.2^48^ with choosing a random beginning tree and running the chains for 4 million for ITS and also combined sequences of ITS and TEF-1-α or ITS and BT. After casting off burn-in samples, the residual samples were reserved for additional analyses. The Markov Chain Monte Carlo (MCMC) method within a Bayesian framework was utilized to assess the posterior probabilities of the phylogenetic trees^49^ by 50% majority rule. The obtained phylogenetic tree was visualized via Dendroscope V.3.2.8^50^.

### Pathogenicity test on apple trees

Pathogenicity test was also carried out on 2-year-old apple trees under field conditions (research farm of agriculture faculty, Tarbiat Modares University) on 26 May 2013. The stems of 2-year-old apple trees (cv. Golden Delicious) were surface-sterilized using 70% ethanol. For inoculation, the stems were wounded in the middle part by removing the cortex (5 mm diameter) with a sterile cork borer. Instantly a mycelial plug (5 mm diameter) of 5-day-old culture was placed on the wound, then wrapped using Parafilm. The control was inoculated with a PDA plug. CL and also CP/SP ratios were measured 6 months after inoculation. The experiment was conducted in RCBD with three replications.

### Disease progress curve

To measure AUDPC, CL was measured at 13-time points with 2 weeks intervals during the pathogenicity period on the apple trees. Local daily temperatures from 26 May to 24 November 2013 were provided by the synoptic weather station of Chitgar-Tehran-Iran. AUDPC was calculated for CL every 2 weeks until 6 months after inoculation according to Eq. (1)^51^.

1 $$\mathrm{AUDPC}={\sum }_{\mathrm{i}=1}^{\mathrm{n}}(\frac{{\mathrm{x}}_{\mathrm{i}}+{\mathrm{x}}_{\mathrm{i}+1}}{2} )\times \left({\mathrm{t}}_{\mathrm{i}+1}-{\mathrm{t}}_{\mathrm{i}}\right),$$

where n denotes the number of measurements, x is CL at each measurement, t signifies the number of days between measurements.

### Fungal growth in different temperatures

The experiment was conducted based on RCBD with factorial arrangement, two factors containing fungal isolate type with 13 levels (OU4, OU12, KH27, KH40, SS65, SH86, SS98, SS100, SK109, KO120, D131, D134, D139) and temperature with seven levels (10, 15, 20, 25, 30, 35, and 40) and three replicates. One agar plug (5 mm diameter) containing mycelia of each fungal isolate out of the actively growing colony edge per replication and was cultured individually in individual Petri dishes (100 × 15 mm) containing 20 mL PDA. Mycelial radial growth was measured at 24 and 72 h after culturing. Mean daily radial growth (MDRG) was calculated by Eq. (2)^52^.

2 $$MDRG=\left(\frac{{R}_{1}-{R}_{2}}{2}\right) \times 100,$$

where R_1_ is the radius of the pathogen colony after 72 h, and R_2_ signifies the radius of the pathogen colony after 24 h.

### Statistical analysis

The hypothesis of normality and equal variance were tested, and data transformation was performed using square root and log base 10 for the detached branch and 2-year-old apple tree experiments, respectively. Conventional parametric statistics were applied for the analysis. The data was statistically analyzed by using SAS (SAS 9.1) and SPSS (SPSS 15.0). ANOVA was conducted by GLM statistical method and means comparison was done by least significant difference (LSD) test. GraphPad Prism (GraphPad Prism 5) software was used for making graphs.

### Ethical approval

All authors approve Ethics and consent for participation and publication. All authors of the manuscript have read and agreed to its content and are accountable for all aspects of the accuracy and integrity of the manuscript in accordance with ICMJE criteria. That the article is original, has not already been published in a journal, and is not currently under consideration by another journal.

## Supplementary Information

Supplementary Information.

## Data Availability

The dataset supporting the conclusions of this article is included in the article and Supplementary information.
