# Author Correction: Fungal canker agents in apple production hubs of Iran

**DOI:** 10.1038/s41598-021-02855-2

**Published:** 2021-11-30

**Authors:** Abbas Nourian, Mina Salehi, Naser Safaie, Fatemeh Khelghatibana, Jafar Abdollahzadeh

**Affiliations:** 1 Department of Plant Pathology, Faculty of Agriculture, Tarbiat Modares University, Tehran, Iran; 2 Department of Plant Breeding and Genetics, Faculty of Agriculture, Tarbiat Modares University, Tehran, Iran; 3 Iranian Research Institute of Plant Protection, Agricultural Research, Education and Extension Organization (AREEO), Tehran, Iran; 4 Department of Plant Protection, Faculty of Agriculture, University of Kurdistan, Sanandaj, Iran

Correction to: *Scientific Reports* https://doi.org/10.1038/s41598-021-02245-8, published online 22 November 2021

The original version of this Article contained an error in Figure 11 where “*Eutypa* cf. *lata*” was incorrectly given as “*Eutypalata*” in the key. The original Figure 11 and accompanying legend appear below.

The original Article has been corrected.

**Figure 11:**
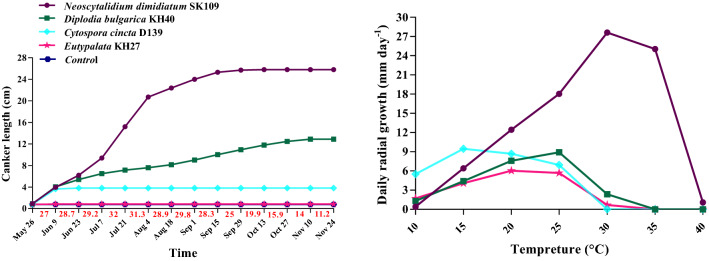
Canker progress curves of different species in the pathogenicity test on 2-year-old apple trees, Temperature is in red, and effect of temperature on daily radial growth of different species on potato dextrose agar. Average values (triplicate) are given.

