# Supplementary material for: Fungal canker agents in apple production hubs of Iran
Source: Sci Rep. 2021 Nov 22;11:22646. doi: 10.1038/s41598-021-02245-8 (PMC8608896; doi:10.1038/s41598-021-02245-8)
Supplement: Supplementary file 1 — Supplementary Information. [file 41598_2021_2245_MOESM1_ESM.docx]

**Supplementary Information**

**Canker casual agents in apple production hubs in Iran**

**Abbas Nourian^1^, Mina Salehi^2^, Naser Safaie^1*^, Fatemeh Khelghatibana^3^ and Jafar Abdollahzadeh^4^**

^1^Department of Plant Pathology, Faculty of Agriculture, Tarbiat Modares University, Tehran, Iran. ^2^Department of Plant Breeding and Genetics, Faculty of Agriculture, Tarbiat Modares University, Tehran. Iranian Research Institute of Plant Protection, Agricultural Research, Education and Extension Organization (AREEO), Tehran, Iran. ^4^Department of Plant Protection, Faculty of Agriculture, University of Kurdistan.

^*^Correspondence to [nsafaie@modares.ac.ir](mailto:nsafaie@modares.ac.ir)

| Table S1. Morphological characters of fungal strains isolated from the trees displaying canker symptoms in apple production hubs of Iran and isolates selected (green) for pathogenicity test on detached branches | | | | | | |
| --- | --- | --- | --- | --- | --- | --- |
|  | Isolate name | Province | Collection site | Colony shape | Colony color | Pathogenicity |
| 1 | OU 1-1 | West Azerbaijan – Urmia | Ghafar Behi village | Flat | White |  |
| 2 | OU 2-1 |  |  |  |  |  |
| 3 | **OU 3-1** |  |  |  |  | _ |
| 4 | **OU 4-1** |  |  |  |  | + |
| 5 | **OU 5-1** |  |  |  |  | _ |
| 6 | OU 6-1 |  |  |  |  |  |
| 7 | OU 7-1 |  |  |  |  |  |
| 8 | OU 8-1 |  |  |  |  |  |
| 9 | OU 8-2 |  |  |  |  |  |
| 10 | **OU 8-3** |  |  |  |  | _ |
| 11 | **OU 9-1** |  |  |  |  | _ |
| 12 | **OU 9-2** |  |  |  |  | _ |
| 13 | **OU 10-1** |  | Tala Tappeh village |  |  | _ |
| 14 | OU 10-2 |  |  |  |  |  |
| 15 | **OU 10-3** |  |  |  |  | _ |
| 16 | OU 11-1 |  |  |  |  |  |
| 17 | OU 12-1 |  |  | Fluffy | Olivaceous |  |
| 18 | **OU 12-2** |  |  |  |  | + |
| 19 | **OU 13-1** |  | Shur Kand village | Flat | White | _ |
| 20 | **OU 14-1** |  |  |  |  | _ |
| 21 | OU 15-1 |  | Qaraguz-e Hajji Baba village |  |  |  |
| 22 | **OU 15-2** |  |  |  |  | _ |
| 23 | OU 16-1 |  |  |  |  |  |
| 24 | OU 17-1 |  |  |  |  |  |
| 25 | **OU 17-2** |  |  |  |  | _ |
| 26 | OU 18-1 |  |  |  |  |  |
| 27 | OU 19-1 |  |  |  |  |  |
| 28 | **OU 19-2** |  |  |  |  | _ |
| 29 | **OU 20-1** |  |  |  |  | _ |
| 30 | OU 21-1 |  |  |  |  |  |
| 31 | **OU 22-1** |  |  |  |  | _ |
| 32 | **OU 22-2** |  |  |  |  | _ |
| 33 | KH 23-1 | West Azerbaijan – Khoy | Firuraq city |  |  |  |
| 34 | **KH 24-1** |  |  |  |  | _ |
| 35 | KH 25-1 |  |  | Semi-fluffy | White |  |
| 36 | **KH 26-1** |  |  |  |  | _ |
| 37 | KH 27-1 |  |  |  |  |  |
| 38 | **KH 27-2** |  |  |  |  | + |
| 39 | KH 28-1 |  |  | Flat | White |  |
| 40 | **KH 28-2** |  |  |  |  | _ |
| 41 | KH 29-1 |  |  |  |  |  |
| 42 | KH 30-1 |  |  |  |  |  |
| 43 | KH 31-1 |  |  |  |  |  |
| 44 | KH 32-1 |  |  |  |  |  |
| 45 | KH 32-2 |  |  |  |  |  |
| 46 | **KH 32-3** |  |  |  |  | _ |
| 47 | KH 33-1 |  |  |  |  |  |
| 48 | **KH 34-1** |  |  |  |  | _ |
| 49 | KH 35-1 |  |  |  |  |  |
| 50 | **KH 35-2** |  |  |  |  | _ |
| 51 | **KH 36-1** |  |  |  |  | _ |
| 52 | KH 37-1 |  |  | Fluffy | Olivaceous |  |
| 53 | **KH 37-2** |  |  |  |  | _ |
| 54 | **KH 37-3** |  |  | Flat | White | _ |
| 55 | KH 38-1 |  |  |  |  |  |
| 56 | **KH 38-2** |  |  |  |  | _ |
| 57 | KH 39-1 |  |  |  |  |  |
| 58 | KH 40-1 |  | Firuraq city- Zaviyeh-e Hasan Khan village |  |  |  |
| 59 | **KH 40-2** |  |  |  |  | + |
| 60 | **KH 41-1** |  |  | Flat | White | _ |
| 61 | **KH 42-1** |  |  |  |  | _ |
| 62 | **S 43-1** | West Azerbaijan- Salmas | Vardan village |  |  | _ |
| 63 | **S 44-1** |  |  |  |  | _ |
| 64 | S 45-1 |  |  |  |  |  |
| 65 | **S 45-2** |  |  |  |  | _ |
| 66 | S 46-1 |  |  |  |  |  |
| 67 | **S 46-2** |  |  |  |  | _ |
| 68 | **S 47-1** |  |  |  |  | _ |
| 69 | S 48-1 |  |  |  |  |  |
| 70 | S 49-1 |  |  |  |  |  |
| 71 | **S 50-1** |  |  |  |  | _ |
| 72 | **S 51-1** |  | Tazeh Shahr city |  |  | _ |
| 73 | **S 51-2** |  |  |  |  | _ |
| 74 | S 52-1 |  |  |  |  |  |
| 75 | S 53-1 |  |  |  |  |  |
| 76 | S 54-1 |  |  |  |  |  |
| 77 | **S 55-1** |  |  |  |  | _ |
| 78 | **S 56-1** |  |  |  |  | _ |
| 79 | S 57-1 |  |  |  |  |  |
| 80 | **S 57-2** |  |  |  |  | _ |
| 81 | S 58-1 |  |  |  |  |  |
| 82 | S 59-1 |  |  |  |  |  |
| 83 | S 59-2 |  |  |  |  |  |
| 84 | **S 59-3** |  |  |  |  | _ |
| 85 | **S 60-1** |  |  |  |  | _ |
| 86 | S 61-1 |  |  |  |  |  |
| 87 | S 61-2 |  |  |  |  |  |
| 88 | **S 61-3** |  |  |  |  | _ |
| 89 | SS 62-1 |  |  |  |  |  |
| 90 | SS 63-1 |  |  |  |  |  |
| 91 | SS 64-1 |  |  |  |  |  |
| 92 | **SS 65-1** | Isfahan – Semirom | Kharab |  |  | + |
| 93 | SH 66-1 |  |  |  |  |  |
| 94 | SH 67-1 |  |  |  |  |  |
| 95 | SH 68-1 |  |  |  |  | _ |
| 96 | SH 69-1 |  |  |  |  |  |
| 97 | SH 70-1 |  |  |  |  |  |
| 98 | SH 71-1 |  | Hana city |  |  |  |
| 99 | SH 72-1 |  |  |  |  |  |
| 100 | **SH 73-1** |  |  |  |  | _ |
| 101 | SH 74-1 |  |  |  |  |  |
| 102 | SH 75-1 |  |  |  |  |  |
| 103 | SH 76-1 |  |  |  |  |  |
| 104 | SH 77-1 |  |  |  |  | _ |
| 105 | SH 78-1 |  |  |  |  |  |
| 106 | SH 79-1 |  |  |  |  |  |
| 107 | **SH 79-2** |  |  |  |  | _ |
| 108 | **SH 80-1** |  |  |  |  | _ |
| 109 | SH 81-1 |  |  |  |  |  |
| 110 | SH 82-1 |  |  |  |  |  |
| 111 | SH 83-1 |  |  |  |  |  |
| 112 | **SH 84-1** |  |  |  |  | _ |
| 113 | **SH85-1** |  |  |  |  | _ |
| 114 | **SH 85-2** |  |  |  |  | _ |
| 115 | **SH 86-1** |  |  |  |  | + |
| 116 | SH 87-1 |  |  |  |  |  |
| 117 | SH 88-1 |  | Tale Robah |  |  |  |
| 118 | SH 89-1 |  |  |  |  |  |
| 119 | SH 90-1 |  |  |  |  |  |
| 120 | SH 91-1 |  |  |  |  |  |
| 121 | **SS 92-1** |  |  |  |  | _ |
| 122 | SS 93-1 |  |  |  |  |  |
| 123 | **SS 94-1** |  |  |  |  | _ |
| 124 | **SS 95-1** |  |  |  |  | _ |
| 125 | SS 96-1 |  |  |  |  |  |
| 126 | SS 97-1 |  |  |  |  |  |
| 127 | **SS 98-1** |  |  |  |  | + |
| 128 | SS 99-1 |  |  |  |  |  |
| 129 | **SS 100-1** |  |  |  |  | + |
| 130 | SS 101-1 |  |  |  |  |  |
| 131 | **SS 102-1** |  |  |  |  | _ |
| 132 | SK 103-1 |  | Komeh city |  |  |  |
| 133 | SK 104-1 |  |  |  |  |  |
| 134 | **SK 105-1** |  |  |  |  | _ |
| 135 | **SK 106-1** |  |  |  |  | _ |
| 136 | SK 107-1 |  |  |  |  |  |
| 137 | SK 108-1 |  |  |  |  |  |
| 138 | **SK 109-1** |  |  |  |  | _ |
| 139 | **SK 109-2** |  |  | Flat and fluffy | Dark | + |
| 140 | SK 110-1 |  |  | Flat | White |  |
| 141 | **SK 110-2** |  |  |  |  | _ |
| 142 | SK 111-1 |  |  | Flat and fluffy | Dark |  |
| 143 | **SK 111-2** |  |  |  |  | _ |
| 144 | SK 112-1 |  |  | Flat | White |  |
| 145 | SK 113-1 |  |  |  |  |  |
| 146 | **SK 114-1** |  |  |  |  | _ |
| 147 | **SK 115-1** |  |  |  |  | _ |
| 148 | **SK 116-1** |  |  |  |  | _ |
| 149 | **SK 117-1** |  |  | Flat and fluffy | Dark | _ |
| 150 | **SK 117-2** |  |  | Flat | White | _ |
| 151 | KO 118-1 |  |  | Flat and fluffy | Dark |  |
| 152 | KO 119-1 |  |  |  |  |  |
| 153 | KO 120-1 | Isfahan- Khomeyni Shahr | Kooshk city |  |  |  |
| 154 | **KO 120-2** |  |  |  |  | + |
| 155 | KO 121-1 |  |  |  |  |  |
| 156 | **KO 122-1** |  |  |  |  | _ |
| 157 | KHO 123-1 |  | Khomeyni Shahr city |  |  | _ |
| 158 | KHO 124-1 |  |  |  |  |  |
| 159 | **KHO 125-1** |  |  |  |  | _ |
| 160 | KHO 126-1 |  |  |  |  |  |
| 161 | D 127-1 | Tehran – Damavand | Absard city | Fluffy | Olive white |  |
| 162 | **D 127-2** |  |  |  |  | _ |
| 163 | D 128-1 |  |  | Flat | White |  |
| 164 | **D 129-1** |  |  |  |  | _ |
| 165 | D 130-1 |  |  |  |  |  |
| 166 | **D 131-1** |  |  |  |  | + |
| 167 | D 132-1 |  |  |  |  |  |
| 168 | **D 132-2** |  |  |  |  | _ |
| 169 | **D 133-1** |  | Ayneh Varzan village |  |  | _ |
| 170 | **D 134-1** |  |  |  |  | + |
| 171 | D 135-1 |  |  |  |  |  |
| 172 | D 136-1 |  |  |  |  |  |
| 173 | **D 137-1** |  | Sarbandan village |  |  | _ |
| 174 | **D 137-2** |  |  |  |  | _ |
| 175 | D 138-1 |  |  |  |  |  |
| 176 | **D 139-1** |  |  |  |  | + |
| 177 | D 140-1 |  |  |  |  |  |
| 178 | **D 141-1** |  |  |  |  | _ |
| 179 | D 142-1 |  |  |  |  |  |
| 180 | D 143-1 |  |  |  |  |  |
| 181 | D 144-1 |  |  |  |  |  |
| 182 | **D 144-2** |  |  |  |  | _ |
| 183 | D 145-1 |  |  |  |  |  |
| 184 | D 146-1 |  |  |  |  |  |
| 185 | **146-2** |  |  |  |  | _ |
| 186 | **D 147-1** |  |  |  |  | _ |
| 187 | D 148-1 |  |  |  |  |  |
| 188 | D 149-1 |  |  |  |  |  |
| 189 | **D 150-1** |  |  | Fluffy | Snow white | _ |

| **Table S2.** Frequency of different fungal morphotypes isolated from tree displaying canker symptoms and selected for pathogenicity test on detached branches in the different collection sites | | |
| --- | --- | --- |
| **Selected fungal strains** | **Isolated fungal strains** | **Collection site** |
| 6 flat and white | 12 flat and white | Ghafar Behi village |
| 2 flat and white  1 fluffy and olivaceous | 4 flat and white  2 fluffy and olivaceous | Tala Tappeh village |
| 2 flat and white | 2 flat and white | Shur Kand village |
| 6 flat and white | 12 flat and white | Qaraguz-e Hajji Baba village |
| 8 flat and white  2 semi-fluffy and white  1 fluffy and olivaceous | 19 flat and white  4 semi-fluffy and white  2 fluffy and olivaceous | Firuraq city |
| 1 fluffy and olivaceous  2 flat and white | 2 fluffy and olivaceous  2 flat and white | Zaviyeh-e Hasan Khan village |
| 6 flat and white | 10 flat and white | Vardan village |
| 8 flat and white | 20 flat and white | Tazeh Shahr city |
| 2 flat and white | 6 flat and white | Kharab |
| 8 flat and white | 19 flat and white | Hana city |
| 6 flat and white | 15 flat and white | Tale Robah |
| 8 flat and white  3 dark, flat and fluffy | 15 flat and white  6 dark, flat and fluffy | Komeh city |
| 2 dark, flat and fluffy | 4 dark, flat and fluffy | Kooshk city |
| 2 dark, flat and fluffy | 4 dark, flat and fluffy | Khomeyni Shahr city |
| 1 fluffy and olive white  3 flat and white | 2 fluffy and olive white  6 flat and white | Absard city |
| 2 flat and white | 4 flat and white | Ayneh Varzan village |
| 7 flat and white  1 fluffy and snow white | 16 flat and white  1 fluffy and snow white | Sarbandan village |

| Table S3. Means comparison for CL, CL/SL, CP/SP indices and AUDPC CL in the pathogenicity/virulence on apple detached branches and trees | | | | | | | |
| --- | --- | --- | --- | --- | --- | --- | --- |
| Isolates | detached branch | | |  | two-year-old apple trees | | |
|  | **CL (cm)** | **CL/SL** | **CP/SP** |  | **CL (cm)** | **AUDPC CL** | **CP/SP** |
| OU4 | 17.8ab | 0.89ab | 1a |  | 1.8f | 303.1g | 0.39d |
| OU12 | 18.6ab | 0.93ab | 1a |  | 9.9c | 1383.9b | 0.54c |
| KH27 | 8.6e | 0.43e | 0.59b |  | 0.9g | 149.1h | 0.25e |
| KH40 | 20a | 1a | 1a |  | 12.9b | 1574.4b | 0.66b |
| SS65 | 16c | 0.8c | 1a |  | 2.1ef | 349.8fg | 0.4d |
| SH86 | 14.4c | 0.72c | 1a |  | 2.4ef | 415.8ef | 0.38d |
| SS98 | 16.4bc | 0.82bc | 1a |  | 2.4ef | 425.6ef | 0.38d |
| SS100 | 10.4d | 0.52d | 0.98a |  | 2.1ef | 350.7fg | 0.36d |
| SK109 | 18.8ab | 0.94ab | 0.98a |  | 25.8a | 3426.9a | 1a |
| KO120 | 10de | 0.5de | 0.58b |  | 3.6d | 489.3de | 0.5c |
| D131 | 15.8c | 0.79c | 1a |  | 3.0de | 520.1d | 0.55c |
| D134 | 8.6e | 0.43e | 0.93a |  | 3.1de | 546.7cd | 0.52c |
| D139 | 14.8c | 0.74c | 1a |  | 3.8d | 652.3c | 0.53c |
| NP | 2.2f | 0.11f | 0.18c |  | - | - | - |
| Control | 1.2f | 0.06f | 0.15c |  | 0.9g | 131.9h | 0.21e |
| CL; canker length, CL/SL ratio; canker length/stem length, CP/SP; canker perimeter/stem perimeter ratio, AUDPC CL; Area under disease progress curve calculated for canker length, NP; non-pathogenic isolates displayed no symptom on apple detached branches.  Means followed by the same letter are not significantly diﬀerent according to LSD at 0.05 probability level. | | | | | | | |

| **Table S4.** Progress of apple canker caused by *Cytospora cincta* (OU4, SS65, SH86, SS98, SS100, D131, D134 and D139), *Diplodia bulgarica* (OU12 and KH40), *Neoscytalidium dimidiatum* (SK109 and KO120) and *Eutypa* cf. *lata* (KH27) in pathogenicity test on two-year-old apple trees | | | | | | | | | | | | | |
| --- | --- | --- | --- | --- | --- | --- | --- | --- | --- | --- | --- | --- | --- |
| **Isolate** | **OU4** | **OU12** | **KH27** | **KH40** | **SS65** | **SH86** | **SS98** | **SS100** | **SK109** | **KO120** | **D131** | **D134** | **D139** |
| **Time** |  |  |  |  |  |  |  |  |  |  |  |  |  |
| **May 26** | 0.8 | 0.8 | 0.8 | 0.9 | 0.8 | 0.8 | 0.8 | 0.8 | 0.9 | 0.9 | 0.8 | 0.8 | 0.8 |
| **Jun 9** | 1.7 | 3.9 | 0.9 | 4.0 | 1.7 | 1.7 | 2.4 | 1.5 | 3.9 | 1.5 | 2.8 | 3 | 3.6 |
| **Jun 23** | 1.8 | 5.0 | 0.9 | 5.4 | 2.1 | 2.4 | 2.4 | 2.1 | 6.2 | 1.8 | 3.0 | 3.1 | 3.8 |
| **Jul 7** | 1.8 | 6.0 | 0.9 | 6.5 | 2.1 | 2.4 | 2.4 | 2.1 | 9.4 | 2.0 | 3.0 | 3.1 | 3.8 |
| **Jul 21** | 1.8 | 6.8 | 0.9 | 7.2 | 2.1 | 2.4 | 2.4 | 2.1 | 15.2 | 2.1 | 3.0 | 3.1 | 3.8 |
| **Aug 4** | 1.8 | 7.1 | 0.9 | 7.6 | 2.1 | 2.4 | 2.4 | 2.1 | 20.7 | 2.1 | 3.0 | 3.1 | 3.8 |
| **Aug 18** | 1.8 | 7.8 | 0.9 | 8.2 | 2.1 | 2.4 | 2.4 | 2.1 | 22.4 | 2.3 | 3.0 | 3.1 | 3.8 |
| **Sep 1** | 1.8 | 8.6 | 0.9 | 9.0 | 2.1 | 2.4 | 2.4 | 2.1 | 24.0 | 3.0 | 3.0 | 3.1 | 3.8 |
| **Sep 15** | 1.8 | 9.0 | 0.9 | 10.0 | 2.1 | 2.4 | 2.4 | 2.1 | 25.3 | 3.5 | 3.0 | 3.1 | 3.8 |
| **Sep 29** | 1.8 | 9.6 | 0.9 | 10.9 | 2.1 | 2.4 | 2.4 | 2.1 | 25.7 | 3.6 | 3.0 | 3.1 | 3.8 |
| **Oct 13** | 1.8 | 9.9 | 0.9 | 11.8 | 2.1 | 2.4 | 2.4 | 2.1 | 25.8 | 3.6 | 3.0 | 3.1 | 3.8 |
| **Oct 27** | 1.8 | 9.9 | 0.9 | 12.5 | 2.1 | 2.4 | 2.4 | 2.1 | 25.8 | 3.6 | 3.0 | 3.1 | 3.8 |
| **Nov 10** | 1.8 | 9.9 | 0.9 | 12.9 | 2.1 | 2.4 | 2.4 | 2.1 | 25.8 | 3.6 | 3.0 | 3.1 | 3.8 |
| **Nov 24** | 1.8 | 9.9 | 0.9 | 12.9 | 2.1 | 2.4 | 2.4 | 2.1 | 25.8 | 3.6 | 3.0 | 3.1 | 3.8 |

| **Table S5.** Average temperatures (°C) of different months in different counties | | | | | | | |
| --- | --- | --- | --- | --- | --- | --- | --- |
| **Moth** | **April** | **May** | **June** | **July** | **August** | **September** | **October** |
| **County** |  |  |  |  |  |  |  |
| **Tehran- Damavand** | 11.3 | 16.7 | 23.2 | 25.8 | 24.2 | 19.2 | 11.9 |
| **West Azerbaijan - Khoy** | 11.25 | 17.4 | 22.3 | 26.9 | 25.2 | 20.3 | 13.6 |
| **West Azerbaijan - Urmia** | 11.6 | 15.4 | 20.8 | 24.6 | 22.8 | 18.3 | 11.7 |
| **Isfahan - Semirom** | 11.1 | 15.6 | 20.4 | 23.2 | 22.3 | 17.3 | 12.1 |
| **Isfahan –Khomeyni Shahr** | 17.4 | 23.1 | 29.1 | 31.7 | 29.8 | 25.7 | 19.6 |

**Fig. S1.** Canker progress curves of *Cytospora cincta* (OU4, SS65, SH86, SS98, SS100, D131 and D134), *Diplodia bulgarica* (OU12), *and Neoscytalidium dimidiatum* (KO120) in the pathogenicity test on two-year-old apple trees. Temperature is in red.

**Fig. S2.** Effect of temperature on daily radial growth of *Cytospora cincta* (OU4, SS65, SH86, SS98, SS100, D131 and D134), *Diplodia bulgarica* (OU12), *Neoscytalidium dimidiatum* (KO120) species on potato dextrose agar.
